# Supplementary material for: Anti-Allergic Effect of 3,4-Dihydroxybenzaldehyde Isolated from Polysiphonia morrowii in IgE/BSA-Stimulated Mast Cells and a Passive Cutaneous Anaphylaxis Mouse Model
Source: Mar Drugs. 2022 Feb 10;20(2):133. doi: 10.3390/md20020133 (PMC8875385; doi:10.3390/md20020133)
Supplement: Supplementary file 1 [file marinedrugs-20-00133-s001.zip › marinedrugs-1551933-supplementary.pdf]

Supplementary files

Article,

Anti-allergic effect of 3,4-dihydroxybenzaldehyde isolated from *Polysiphonia morrowii* in IgE/BSA-stimulated mast cells and a passive cutaneous anaphylaxis mouse model

Eun-A Kim [1,+] , Eui-Jeong Han [2,3,+], Junseong Kim [1], Ilekuttige Priyan Shanura Fernando [4], Jae-Young Oh [5], Kil-Nam Kim [6], Ginnae Ahn [3,4,*], and Soo-Jin Heo [1,7,*]

[1] Jeju Marine Research Center, Korea Institute of Ocean Science & Technology (KIOST), Jeju 63349, Republic of Korea; euna0718@kiost.ac.kr (E.-A. K); junseong@kiost.ac.kr (J. K)

[2] Research Center for Healthcare and Biomedical Engineering, Chonnam National University, Yeosu 59626, Korea; iosu5772@naver.com (E.J.H)

[3] Department of Food Technology and Nutrition, Chonnam National University, Yeosu 59626, Republic of Korea; iosu5772@naver.com (E.J.H)

[4] Department of Marine Bio-Food Sciences, Chonnam National University, Yeosu 59626, Republic of Korea; shanurabru@ jnu.ac.kr (I.P.S.F)

[5] Food Safety and Processing Research Division, National Institute of Fisheries Science, Busan 46083, Republic of Korea; ojy0724@korea.kr (J.-Y.O)

[6] Chuncheon Center, Korea Basic Science Institute (KBSI), Chuncheon 24341, Republic of Korea; knkim@kbsi.re.kr (K.-N.K)

[7] Department of Marine Biology, University of Science and Technology, Daejeon 34113, Republic of Korea; e-mail@e-mail.com

[*] Correspondence: gnahn@jnu.ac.kr (G.A); sjheo@kiost.ac.kr (S.-J.H.); Tel: +82-61-659-7213 (G.A); +82-64-798-6039 (S.-J.H.)

[+] These authors contributed equally to this study

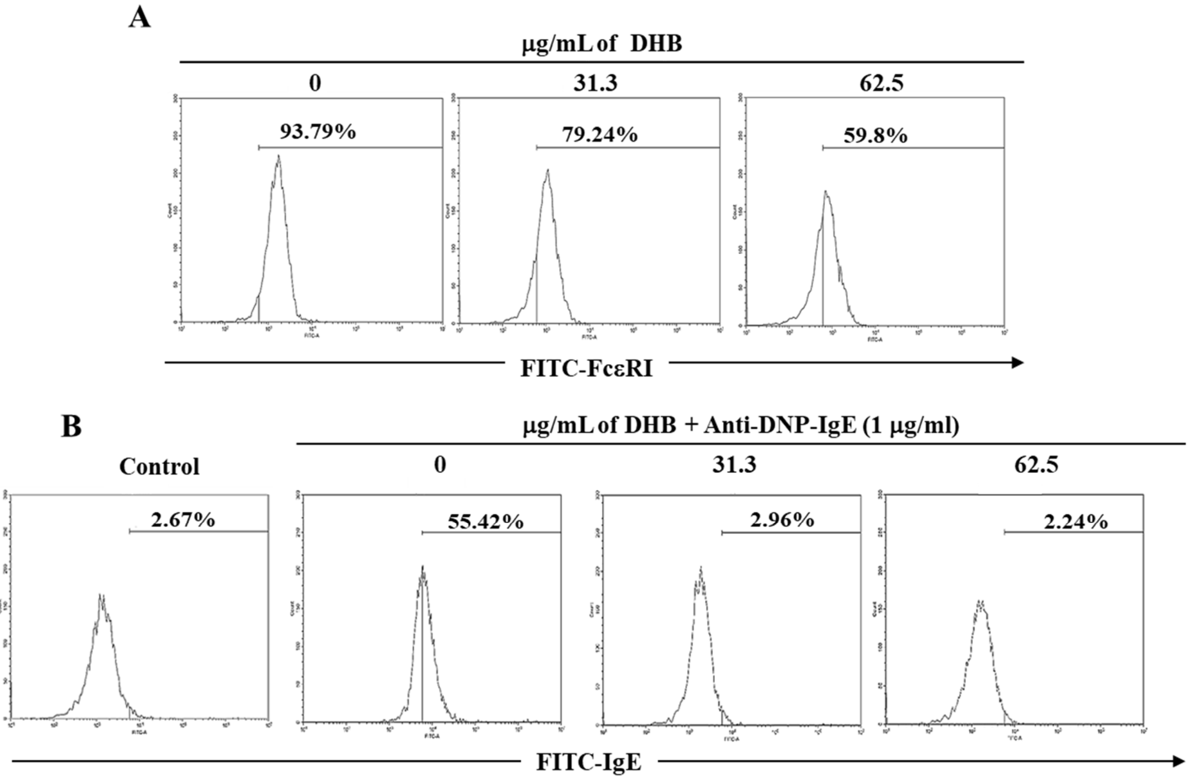

Figure S1. Effects of DHB on FcεRI expression and the crosslinking of IgE to FcεRI expression in IgE/BSA-stimulated BMCMCs. (A) cell surface FcεRI expression and (B) IgE antibody binding to FcεRI in BMCMCs.

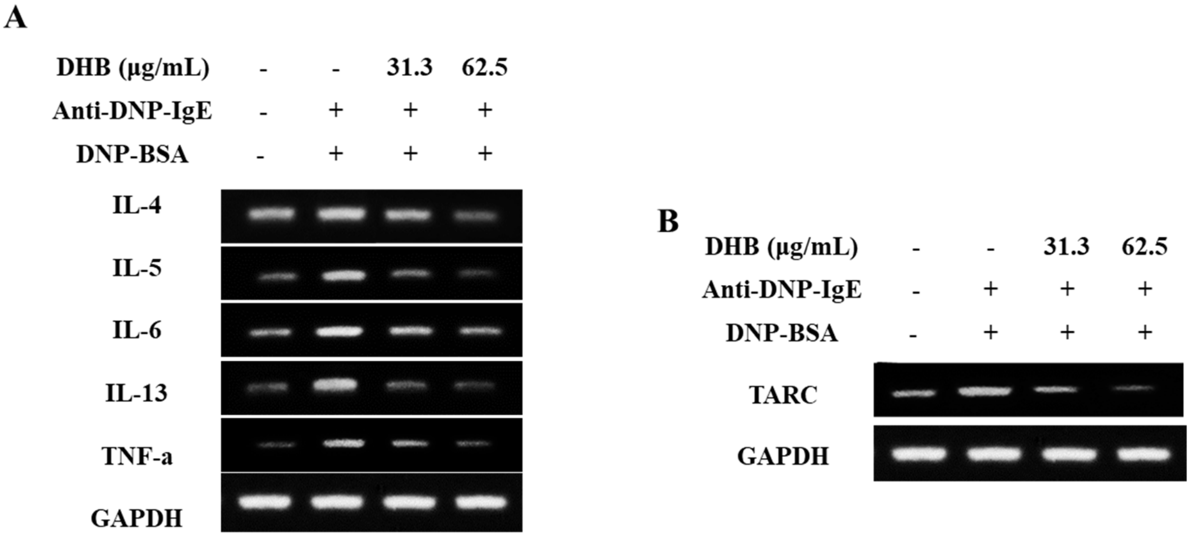

Figure S2. Effects of DHB on the mRNA expression levels of allergic cytokines and chemokine in IgE/BSA-stimulated BMCMCs. The expression levels of (A) cytokines and (B) chemokine using RT-PCR analysis.

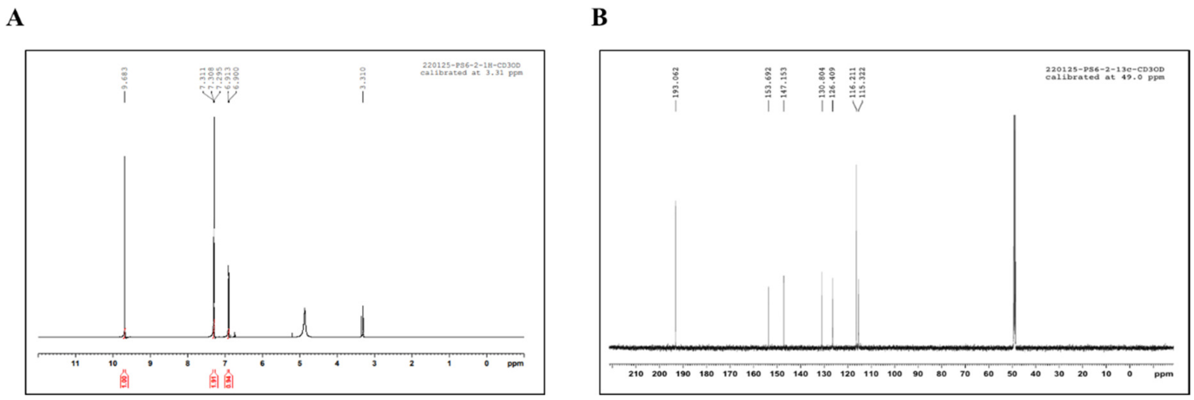

Figure S3. The 1H (A) and 13 C (B) NMR spectrum of DHB.
